# Osteonevus of Nanta Presenting as Nodule over Left Eyebrow

**DOI:** 10.1155/2012/715672

**Published:** 2012-03-26

**Authors:** Girish Kamat, Aneel Myageri, Ravikala Rao

**Affiliations:** Department of Pathology, SDM College of Medical Sciences and Hospital, Manjushree Nagar, Dharwad 580009, India

## Abstract

Osteonevus of Nanta is a rare histopathological finding in which there is an ectopic bone formation in intradermal nevus. Very few cases have been reported so far in the literature. We present a case of osteonevus of Nanta, which occurred as a nodule over the left eyebrow in a 52-year-old female patient. Simple excision proved to be curative in the present case.

## 1. Introduction

Many interesting changes can uncommonly be seen in benign intradermal nevus. The incidental finding of bone formation in intradermal nevus is known as osteonevus of Nanta. Cutaneous bone formation is an uncommon phenomenon in the skin which may be primary or secondary. Historically, primary cases have included entities such as Albright hereditary osteodystrophy, progressive osseous heteroplasia, myositis ossificans progressiva, diaphyseaal aclasis, and osteoma cutis. Secondary bone formation has been reported in variety of lesions such as pilomatricoma, basal cell carcinoma, acne, melanocytic nevi, cellular nevus, cylindroma, and dermatofibroma [1]. 

## 2. Case Presentation

A 52-year-old female patient presented to department of plastic surgery with a pigmented nodule over the left eye brow. A clinical diagnosis of intradermal nevus was made, and the lesion was excised along with adjacent skin.

Grossly, the specimen consisted of elliptical skin with a nodule measuring 0.5 cm in diameter. Cut section showed a brown-colored tumor beneath the skin.

**Figure 1:**
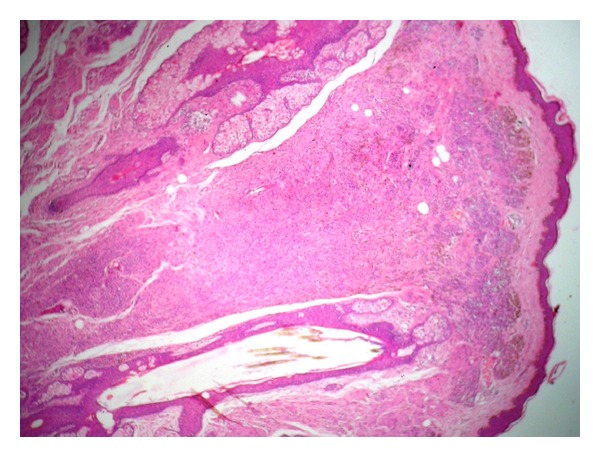
Nests of nevus cells with overlying normal epidermis (H&E, 10x).

**Figure 2:**
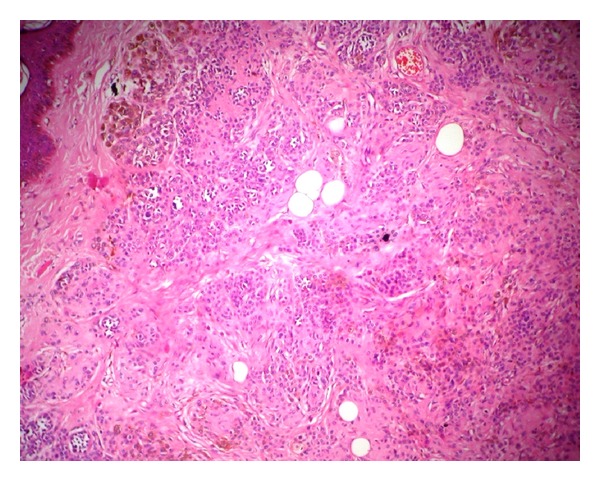
Clusters of nevus cells with melanin pigment (H&E, 40x).

**Figure 3:**
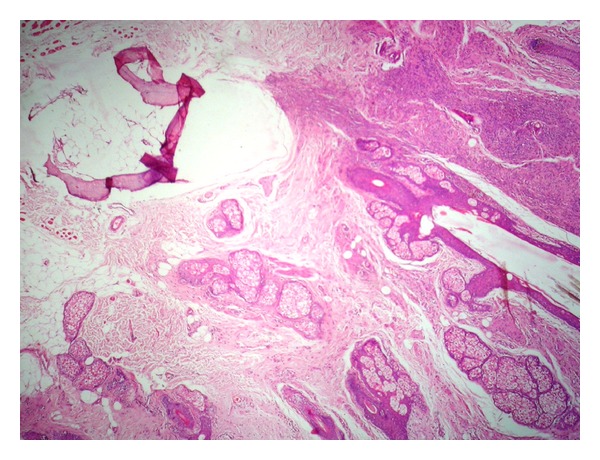
Lamellar bone which is seen adjacent to nevus cells (H&E, 10x).

Microscopy showed normal epidermis overlying nests of nevus cells having large nucleus and scanty eosinophilic cytoplasm (Figure 1). Superficial clusters contained melanin pigment (Figure 2). Few giant cells were also seen. Hair follicles in the vicinity of nevus were dilated and contained dermatophyte spores. Adjacent to nevus a well-circumscribed lamellar bone with fatty marrow at the centre was seen (Figure 3). Therefore the diagnosis of osteonevus of Nanta (or osseous metaplasia in benign intradermal melanocytic nevus) was made.

## 3. Discussion

Several interesting changes may be seen in nevi. They include the incidental finding of amyloid or of bone; the concurrence of psoriasis; epidermal spongiosis producing a clinical eczematous halo-Meyerson's nevus; increased amount of elastic tissue; cystic dilatation of related hair follicles; nodular myxoid change; psammoma body formation; focal epidermal necrosis; an incidental molluscum contagiosum; an associated trichoepithelioma, basal cell carcinoma, syringoma, or sweat duct proliferation [2]. Overall, cases of osteonevus of Nanta are rarely reported in English literature. But in reports of large series of skin osteomas, they represent 20% of skin lesions with bone formation [3]. Considering the scarcity of published reports, this is somewhat surprising. Bone formation is usually present at the base of the melanocytic lesions, and these lesions tend to be located in the upper part of body, as in our case.

In addition, there is a higher incidence of this phenomenon in females. This interesting observation remains largely unexplained. It is well known that osteoblasts have surface receptors for estrogen. Binding of estrogen to these receptors releases cytokines, which results in downregulation of bone resorption. Therefore it can be speculated that the effects of estrogen on osteoblasts together with the lack of osteoclastic activity may explain why these lesions are seen more in female patients, although additional studies are required to confirm this attractive hypothesis [4]. 

## 4. Conclusion

We report a unique case of osseous metaplasia within a benign intradermal nevus, osteonevus of nanta, in a female patient. Clinical appearance of this lesion is similar to ordinary nevus. There is one reported case in the literature of a malignant melanoma arising within an osteonevus of Nanta by Culver and Burgdorf [5]. Therefore one must be aware of this association when they encounter osseous metaplasia in melanocytic lesions, rather than just disregarding this interesting but unusual phenomenon as an unimportant finding.

## References

[1] Philip A, Conlin M, Laura P, Jiminez Q, Rapini R (2002). Osteomas of skin revisited—a clinic pathological review of 74 cases. *The American Journal of Dermatopathology*.

[2] David W (2002). *Skin Pathology*.

[3] Moulin G, Souquet D, Malme B (1991). Pigmented nevus and cutaneous ossification. *Annales de Dermatologie et de Vénéréologie*.

[4] Al-Daraji W (2007). Osteo-nevus of Nanta: an uncommon phenomenon. *Dermatology Online Journal*.

[5] Culver W, Burgdorf WHC (1993). Malignant melanoma arising in a nevus of Nanta. *Journal of Cutaneous Pathology*.

